# One Health Surveillance Codex: promoting the adoption of One Health solutions within and across European countries

**DOI:** 10.1016/j.onehlt.2021.100233

**Published:** 2021-03-05

**Authors:** Matthias Filter, Tasja Buschhardt, Fernanda Dórea, Estibaliz Lopez de Abechuco, Taras Günther, Esther M. Sundermann, Jörn Gethmann, Johanna Dups-Bergmann, Karin Lagesen, Johanne Ellis-Iversen

**Affiliations:** a German Federal Institute for Risk Assessment (BfR), Max-Dohrn-Straße 8–10, 10589 Berlin, Germany; b Department of Disease Control and Epidemiology, National Veterinary Institute, SE-751 89 Uppsala, Sweden; c Friedrich-Loeffler-Institute, Institute of Epidemiology - Federal Research Institute for Animal Health, Südufer 10, 17493 Greifswald-Insel Riems, Germany; d Norwegian Veterinary Institute, Ullevålsveien 68, 0454 Oslo, Norway; e National Food Institute, Danish Technical University (DTU-Food), 2800 Kgs. Lyngby, Denmark

**Keywords:** Knowledge base, Collaboration, Dissemination, Mutual understanding, Data interoperability

## Abstract

Cross-sector communication, collaboration and knowledge exchange are still significant challenges for practical adoption of the One Health paradigm. To address these needs the “One Health Surveillance Codex” (OHS Codex) was established to provide a framework for the One Health community to continuously share practical solutions (e.g. tools, technical resources, guidance documents and experiences) applicable for national and international stakeholders from different One Health Surveillance sectors. Currently, the OHS Codex provides a number of resources that support the adoption of the OH paradigm in areas linked to the harmonization and interpretation of surveillance data. The OHS Codex framework comprises four high-level “action” principles, which respectively support collaboration, knowledge exchange, data interoperability, and dissemination. These principles match well with priority areas identified in the “Tripartite Guide to Addressing Zoonotic Diseases in Countries” published by WHO, FAO and OIE. Within each of the four principles, the OHS Codex provides a collection of useful resources as well as pointers to success stories for the application of these resources. As the OHS Codex is designed as an open community framework, it will continuously evolve and adapt to the needs of the OH community in the future.

## Highlights

• The One Health Surveillance (OHS) Codex supports the WHO/FAO/OIE Tripartite Guide.
• The resources in the OHS Codex support OHS data interpretation and harmonization.
• The OHS Codex is a community resource supporting the adoption of the OH paradigm.
• The OHS Codex allows sharing of practical experiences from various OH efforts.
• The OHS Codex is an open resource continuously updated by and for the community.

## Graphical abstract

Graphical Abstract

**Figure:**
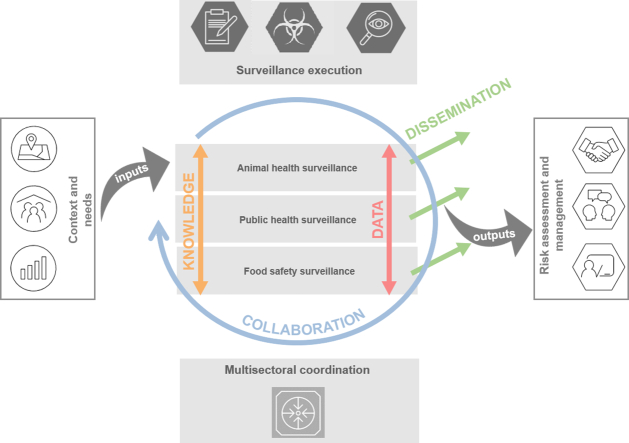

## 1 Introduction

It is widely accepted that there is a fundamental link between threats posed by zoonotic diseases to humans and the health of domestic animals, wildlife and the environment [1]. The One Health (OH) concept is an effort to de-compartmentalise the human health, animal health and environmental sectors for more efficient and sustainable governance of complex health issues [2]. This OH concept also guides the management of risks posed by emerging infectious diseases (EID) where effective global surveillance is considered the essential component for early detection of EIDs [1]. In many European countries environmental science, public health, veterinary health and food safety are well established and have often evolved as independent scientific disciplines with independent national (and international) institutions. Therefore, adopting the OH approach in current sectorial surveillance and monitoring practices is still a significant challenge for developed countries [3].

This paper describes a continuously updateable framework that could help to overcome the many obstacles preventing the broad adoption of the OH paradigm in day-to-day surveillance practice by sharing guidelines, tools, experiences and other useful resources. The proposed framework is called “One Health Surveillance Codex” (OHS Codex) as it aims to serve as a cross-sector, international, but not legally binding resource. The OHS Codex provides solutions that will help to improve cross-sector collaboration, mutual understanding, knowledge exchange and information dissemination. It also includes practical resources that can be applied to improve cross-sector surveillance data communication, exchange and interpretation.

The OHS Codex aligns with related activities, e.g. the WHO/FAO/OIE Tripartite Guide to Addressing Zoonotic Diseases in Countries [4] (or ‘Tripartite Zoonoses Guide’) and the One Health Global Network [5], by adding newly developed resources and lessons learned from practical OH activities carried out in the OH European Joint Programme (OHEJP) project (https://onehealthejp.eu/ [6]).

## 2 European surveillance practices and their OH barriers

In 2018, the members of the OHEJP Joint Integrative Project ORION (https://onehealthejp.eu/jip-orion/ [7]) performed a survey among domain experts to identify the needs in relation to OHS data integration and interpretation. Data for the analysis were collected through interviews, workshops with experts from the OHEJP consortium and an online survey that was sent out to OHEJP member organizations (doi:https://doi.org/10.5281/zenodo.3754468, ORION 2020).

**Fig. 1:**
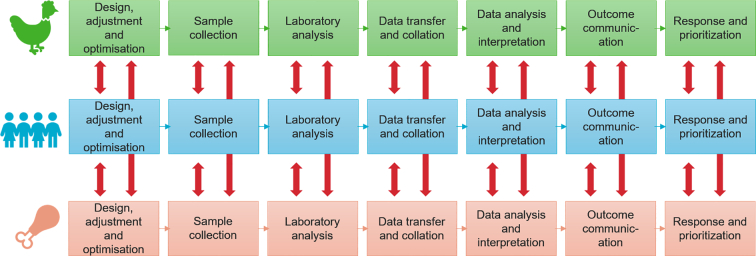
This figure is called “Surveillance Pathway Visualization” as it illustrates the different process steps carried out in animal health, public health and food safety surveillance. The red arrows indicate where surveillance activities could be connected between two or all three sectors to support the OH concept. (For interpretation of the references to colour in this figure legend, the reader is referred to the web version of this article.)

A first outcome from this research was the finding that the surveillance processes in the public health, animal health and food safety sectors can be described as a continuum of activities from planning based on a specific goal, gathering data and data analysis, to the use of the generated information for action, dissemination and feedback. This cycle of activities was called the “Surveillance Pathway Visualization” (see Fig. 1). It was clarified that OH activities, e.g. a country-specific or regional collaboration between animal health, public health and food safety sectors, could be established at any particular point of the pathway.

Another key result of this research was the identification of the following barriers to OH implementation:

• The approaches to adopt the OH concept differ greatly across European countries.
• There is a great heterogeneity between EU countries in the number and type of institutional bodies that are relevant for OH implementation.
• There is the need for more cross-sector collaboration and knowledge exchange, e.g. experience with surveillance in individual sectors exists, but there is sometimes no common vision of what OH surveillance means in practice.
• There are significant deficits in cross-sector communication, e.g. sector-specific terminology is widely used and no resources for terminology mapping or harmonization are available.
• There is the need for a generic approach that allows consistent cross-sector surveillance data interpretation, as this is currently hampered by the intrinsic complexity of established surveillance systems and the lack of standards to describe them (compare Codex Alimentarius [8]).
• Solutions and concepts for providing surveillance data in interoperable formats are missing or need to be further improved and promoted.
• There is a lack of ready-to-use “tools” to apply the OH surveillance concept in practice.

In order to find suitable approaches to address these barriers, the ORION project team worked towards the establishment of an integrated strategy for the collection, interpretation, sharing and dissemination of surveillance data, surveillance information and surveillance knowledge (doi:https://doi.org/10.5281/zenodo.3754468, [9]). In this process, the analysis of existing frameworks that describe the organisation of the different organisational levels of collaboration in OHS systems [2,4,10] helped us to identify three non-exclusive strategies that could be followed to promote OH implementation in routine surveillance practice:

a) surveillance could be continued as before, independently in each sector, but result dissemination should take into account the information needs of other sectors;
b) surveillance activities could be performed jointly by different sectors at specific steps along the surveillance pathway;
c) new surveillance processes could be designed jointly as an integrated OH activity that supports the joint as well as sector-specific objectives, needs and actors.

As discussions progressed among ORION project partners from different OH sectors and countries, it became clear that each country needs to specify its own set of measures to improve OH adoption in the future. Positively, it was concluded that implementation of the OH concept does not depend on shared governance across all sectors, so progress can also be achieved by building on existing, sector-specific governance structures. With the right set of tools and the joint will for collaboration, effective strategies for OH surveillance can be implemented. Thus, the creation of tools and resources that support collaboration, knowledge sharing and data exchange in OH surveillance were identified as a priority development goal. To make such tools and resources available to all surveillance actors at the time of need, it was considered important to create an overarching framework that is capable of disseminating such resources in an easy to maintain manner. This new framework is called the One Health Surveillance Codex (OHS Codex).

## 3 OHS Codex design

**Fig. 2:**
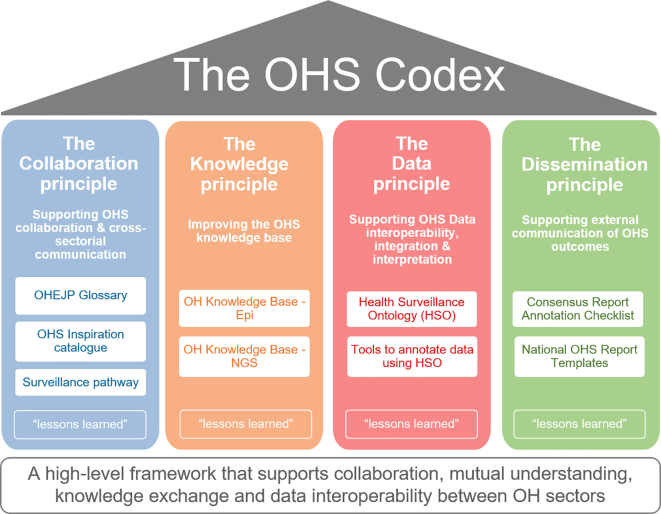
Overview of the OHS Codex framework structured into four principles. The white boxes under each principle include the *Methods* (e.g. solutions, tools and resources) included so far to enhance OHS. The box “*lessons learned”* is a placeholder for whitepapers or articles describing the findings from implementation or usage of *Methods* listed under the corresponding principle.

**Fig. 3:**
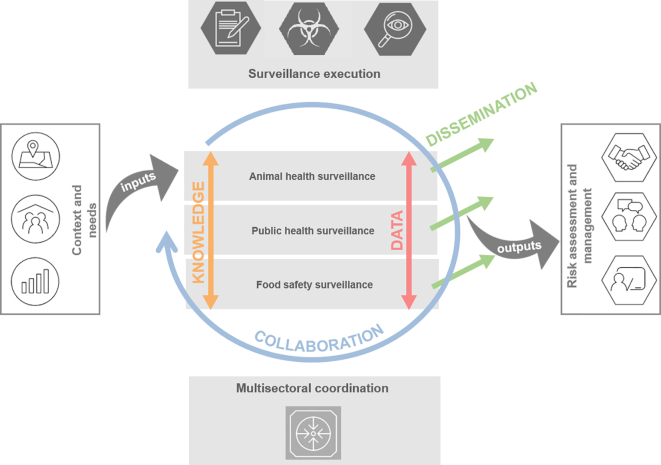
Connection between the elements of the Tripartite Zoonoses Guide [4] and the four principles of the OHS Codex: Knowledge, Data, Dissemination and Collaboration. Coloured arrows indicate the dimensions on which the OHS Codex principles operate. The pictograms are taken from [4].

The OHS Codex was designed as a knowledge resource for researchers and practitioners working in OH related disciplines, like animal or public health officials involved in disease surveillance or risk assessment. Its main purpose is to facilitate efficient exchange of knowledge and sharing of resources, guidelines, tools and experiences promoting the adoption of the OH concept primarily in relation to surveillance data. To provide the OHS Codex with a logical and consistent overarching structure, high-level “principles” were defined, following the example of the Quality Assurance Framework of the European Statistical System [11]. For the OHS Codex, these principles represent specific “action areas” within the OH activity spectrum, and were named “Collaboration”, “Knowledge”, “Data” and “Dissemination” (see Fig. 2). The relationship among these OHS Codex principles and their support to surveillance in practice are illustrated in Fig. 3. This figure highlights that despite the principles “operating” on different abstraction layers, they are still interconnected. In addition, these principles align nicely to the approach outlined in the multisectoral Tripartite Zoonoses Guide [4].

Within the OHS Codex, each principle is defined by a purpose and scope statement. This high-level OHS Codex structure allows the collation and gathering of available or newly developed solutions, tools and resources (called **Methods**) as well as application examples and lessons learned (called **Examples & Lessons learned**) for each of the specific principles (see Fig. 2). The OHS Codex is designed as an extended “table of contents”, listing existing resources with a short summary each. This summary allows end-users to decide if the corresponding resource is of relevance to their specific situation. Further, a link is provided where more detailed information on each Method can be accessed.

The OHS Codex has been implemented as a “Read-the-docs” online document available at: https://oh-surveillance-codex.readthedocs.io/en/latest/index.html [12]. In this way, it can become a community resource that can continuously take up new *Methods* or *Examples & Lessons learned* in each of the principles. Instructions, on how to contribute, are given in the OHS Codex document [13].

## 4 OHS Codex structure

In this section, we briefly introduce each of the four principles that provide the overarching structure to the OHS Codex and illustrate the *Methods* currently listed under each principle. At present, these *Methods* contain primarily resources, tools and *Examples & Lessons learned* that were generated within the ORION project and that address the specific barriers identified during the ORION requirement analysis phase.

The **Collaboration principle** represents the need for positive interaction and communication between actors and is considered the foundation of OH surveillance. Without the willingness and ability to collaborate and communicate, surveillance systems will remain sectorial and fragmented. The principle gathers tools and resources that facilitate the understanding of surveillance across sectors and disciplines, whilst also illustrating that these solutions can be found in environments where collaboration works.

Specific *Methods* listed under the Collaboration principle are the “OHEJP Glossary”, the “Surveillance Pathway Visualization” resource and the “OHS Inspiration catalogue” (for further details see https://oh-surveillance-codex.readthedocs.io/en/latest/2-the-collaboration-principle.html [14]).

The **Knowledge principle** represents the need for mutual scientific understanding and expertise on other sectors' knowledge evolution. Shared knowledge bases highlight the benefits of cross-sector working and enable the transfer of methodologies and expertise between sectors that enhance OHS. The purpose of this principle is therefore to provide surveillance professionals and stakeholders with guidance on available knowledge resources.

To date, the *Methods* in this principle are the “Inventory of Surveillance systems and mathematical methods” and the “Sequencing for Surveillance Handbook” (for further details see https://oh-surveillance-codex.readthedocs.io/en/latest/3-knowledge-principle.html [15]).

The **Data principle** represents the ability to understand, reuse and interpret surveillance data across sectors and disciplines. Without resources that support the proper annotation of surveillance data and their metadata, it will be impossible to correctly find and interpret surveillance data for both humans (e.g. in case of cross-sector interpretation) and machines (interoperability among data systems). In addition, this principle covers activities and resources that promote the formal representation of surveillance knowledge using ontologies. Ontologies can capture domain knowledge in formats that facilitate data processing by a computer. Annotation of data and metadata using these ontologies, even when stored in unstructured formats, can improve their usability inside institutions as well as enable cross-sector reuse.

To date, the *Methods* in the Data principle are the Health Surveillance Ontology and tools for annotation of surveillance data using ontologies (for further details see https://oh-surveillance-codex.readthedocs.io/en/latest/4-the-data-principle.html [16]).

The **Dissemination principle** represents the distribution of surveillance findings to stakeholders and surveillance actors e.g. policy or industry. Often reports from surveillance activities are used as the basis for risk assessments and decision support, e.g. on how to control a risk. The resources provided within this principle are therefore mainly targeted to support the cross-sector harmonized provisioning of metadata in OHS reports. When surveillance outputs and findings are presented following this principle, the decisions made based on these results are likely to also take OH into consideration. To date, the *Methods* listed under this principle are the “One Health Consensus Report Annotation Checklist” (OH-CRAC) and “National OHS Report Templates” (for further details see https://oh-surveillance-codex.readthedocs.io/en/latest/5-the-dissemination-principle.html [17]).

## 5 *Methods* currently listed in the OHS Codex

### 5.1 Collaboration principle

#### 5.1.1 Surveillance pathway visualization

Surveillance is multidisciplinary and a very complex activity. Often, each actor perceives his/her own role as “a surveillance activity”, without a clear view of all the connected steps that generate or consume information from their work. Laboratory experts often interpret detection of a pathogen as surveillance; epidemiologists name collation and analysis of data as surveillance, and decision-makers and their technical advisors also consider their contribution as surveillance. Whereas all actors are doing surveillance, they are usually only part of a process or pathway that has multiple steps, each with their own data, metadata and methods as well as strengths and challenges. The limited perspective of individual surveillance actors is often a source of misunderstandings and collaboration difficulties. When combining experts from multiple sectors, the risk of misunderstandings increases and frequently collaborations are hampered. When the ORION project first brought together multiple actors (microbiologists, epidemiologists, risk analysts, data experts and bioinformaticians) from animal health, food safety and public health such misunderstandings could also be observed. To overcome confusion and prolonged discussions, a schematic illustration of the whole surveillance pathway was needed and jointly developed (Fig. 2). The simplified, schematic visualization of the pathways of each sector ensured that everyone in the project had the same understanding of what surveillance entails. Over time, this ORION Surveillance Pathway Visualization has become a valuable resource that is continuously used in meetings to illustrate, emphasise or align perceptions and solve misunderstandings during discussions.

#### 5.1.2 OHEJP Glossary

OHS documents, e.g. reports and guidelines, rarely contain integrative glossaries with a list of OH related terms and their specific meanings within the context of the given document. The international and cross-sector work within ORION has highlighted that sectors have established their own terminology, which sometimes also involves different interpretations of the same terms. By including a glossary in documents or reports, the author facilitates coherent interpretation of used terms, helping to reduce misunderstandings and misinterpretation of OHS reports. The same is true for verbal communication between different OH actors. An OH glossary is useful to create awareness of these differences in the use of terminology and the interpretation of terms across OH sectors. The “OHEJP Glossary” web portal (accessible via the following website: https://foodrisklabs.bfr.bund.de/ohejp-Glossary/ [18]) is an infrastructure that supports the creation of glossaries. It can be used to find and share available definitions from various sectors, including their references. Among other features, the OHEJP Glossary allows the identification of definitions that are shared between sectors or those that are sector-specific. The established technical infrastructure also supports the collaborative curation and extension processes established for the OHEJP Glossary. The inclusion of glossaries could contribute to better communication, collaboration and (data) comparability between organizations and OHS sectors. An example of a document-specific glossary is given for the present manuscript where all relevant terms are defined in Appendix A.

### 5.2 Knowledge principle

#### 5.2.1 Surveillance inventory

Accessing surveillance data from multiple sectors and multiple member states, for an OH approach to disease management across the EU, can be difficult. The difficulty extends from a lack of accessible information about what pathogens are under surveillance; what data are collected; how the data are analysed; how to access the data or reports; and whether the reports exist in an accessible format. While EFSA and ECDC collect standardized surveillance outcomes from some mandatory or legally founded systems across all member states, several member states carry out additional surveillance. Moreover, many member states publish the results of their surveillance in national reports or national databases, which include additional data to those reported to EFSA or ECDC and might be important for the correct understanding of national surveillance systems. Together, these factors present considerable barriers to using surveillance data from multiple sectors and multiple member states for OH disease management purposes. To overcome these barriers, the ORION project developed an OH surveillance inventory to pull together information on surveillance systems for zoonotic and foodborne pathogens from the food safety, animal health and public health sectors, and from as many member states as possible. The inventory aims to collate the publicly available information on the surveillance systems in existence, the data they collect, and where the data sources can be accessed. The data are stored in a publicly available web application specifically developed to create easy access to the information in a single platform. Furthermore, the application contains intuitive search functions to enable users to effectively access high-level information for their specific needs. For instance, users can identify what surveillance systems exist across all sectors for a pathogen of interest, or where to access official surveillance data for a particular pathogen.

The inventory is designed to be a “living” entity. Currently, it contains information provided by the partners from the ORION project. In the upcoming years, it will be expanded to include information from other EU member states for a more comprehensive picture. To keep the inventory current and relevant it will be updated periodically, and data collection options modified based on feedback from data providers and users.

#### 5.2.2 NGS handbook

Next Generation Sequencing (NGS) is a fairly new and efficient method applied to OH surveillance. In the future, NGS is likely to replace traditional laboratory tests for surveillance purposes. Sequencing of a pathogen's genome can reveal a whole gamut of features relevant for human and animal health, such as antimicrobial resistance and virulence genes. In addition, it can be used to identify genetic differences between closely related isolates and to track these isolates along the human and animal interface. While being a promising method, NGS requires new equipment, skills and a reliable bioinformatics infrastructure for data analysis. The establishment of standard NGS-methods in OHS institutions is therefore challenging. Sequencing requires access to laboratory infrastructure, sequencing instruments and computing infrastructure for storage, analysis and interpretation of data. In addition, trained personnel are needed to operate the equipment and perform bioinformatics analyses.

In order to produce reliable and reproducible NGS results, which are comparable between OH institutions and sectors, it is crucial to establish Standard Operating Procedures (SOPs) for laboratory and bioinformatics methods. To elucidate the NGS practices that are currently in use and to help institutions implement sequencing for surveillance, a One Health Sequencing for Surveillance (SfS) Handbook has been created. Similar to the OHS Codex, the handbook is set up as a community editable Read-the-docs document that also contains guidance on how one can contribute. At present, the SfS Handbook has five sections: a *Guidelines* section with pointers to relevant guidance documents from ECDC, EFSA and other relevant institutions; an *Infrastructure* section, which describes possible options for IT infrastructures and data management; a *Bioinformatics methods* section, which briefly describes methods that are used for data analysis; a *Data analysis toolkit* section, which lists and describes tools that can be used; and an *Interpretation* section which discusses how the analysis results can be used. Provided the SfS Handbook is adopted by the community, it is hoped to become a knowledge exchange hub where implicit knowledge can be made explicit to support the meaningful use of NGS for OH surveillance.

### 5.3 Data principle

#### 5.3.1 Health Surveillance Ontology (HSO)

In the food chain and animal/public health surveillance several steps require processing of data. Differences in data interpretation by humans and lack of interoperability among software systems often prevent timely data exchange between surveillance actors and hinder efficient data integration. An analysis of currently used data workflows in public health, animal health, and food safety institutes from Europe at the beginning of the ORION project allowed us to map the opportunities and challenges in this domain. It became clear that the adoption of harmonized data exchange standards would promote structural interoperability at high levels (such as at the European Union level), but their adoption has a high human resources cost, is time-consuming, and relies on a common understanding of the standards. For the preservation of meaning and context of data across different sectors semantic interoperability is needed. Interoperability solutions explicitly preserving the meaning of data (semantics) take into account that institutions from different sectors might use different metadata coding practices, and that in some cases data and metadata generated are unstructured. Ontologies, in particular, are tools for knowledge modelling that allow humans to describe heterogeneous metadata concepts into a formally structured representation that then can serve as a foundation for semantic interoperability among data systems. The development of an ontology to connect health surveillance sectors was the chosen semantic interoperability solution for two main reasons: 1) the ontology *development* process creates a platform to discuss and document agreements regarding the knowledge needed to process data and generate surveillance information, without the need to directly share data; 2) ontology *adoption* respects data practices of data providers.

The Health Surveillance Ontology (HSO) is a knowledge model for the context of data collection, collation and summarization in OHS. This model was constructed building on already existing terminologies and data standards, that is, reusing their knowledge and maintaining interoperability with data coded by these standards. Ontologies can be developed as interoperable modules – each module is functional for use in one particular context, and additional modules can be added to expand functionality. As a community of surveillance data analysts adopts HSO, the model can be extended or even updated to reflect evolution of knowledge. HSO is currently interoperable with various catalogues in EFSA's Standard Sample Description [19]. Moreover, HSO was developed following the principles of the Open Biological and Biomedical Ontology (OBO) Foundry (http://www.obofoundry.org/ [20]), making it interoperable with several already existing biomedical models of knowledge. The ontology is publicly available at a globally unique and persistent identifier: https://w3id.org/hso [21]. Humans accessing this link via browsers will be referred to a page listing all ontology documentation and additional resources, such as training materials. Software agents pointed to the same address will find the machine-readable codes for the knowledge model (written using the Web Ontology Language - OWL).

#### 5.3.2 Tools for explicit annotation of surveillance data using HSO

HSO is in itself compliant with the FAIR principles of findability, accessibility, interoperability and reuse (https://www.force11.org/group/fairgroup/fairprinciples [22]). It provides the required data annotation model for any data source to attend the FAIR principle of interoperability, as stated in the data principle I2 (“To be interoperable: I2 (meta)data use vocabularies that follow FAIR principles”). If data within datasets are properly annotated with HSO their content becomes accessible (A) and interoperable (I). When HSO is used to annotate metadata about these datasets, they further become findable (F) and reusable (R).

The data annotation process is highly dependent on the data management tools used at each institution. In ORION we have identified that epidemiologists most frequently manipulate and exchange in flat formats, in “.xls”, “.xlsx” or “.csv” formats. For that reason, we have in collaboration with other projects, developed a tool for semantic annotation of data in Excel, and subsequent export of the data in Resource Description Framework (RDF) format, a popular information exchange standard for data interchange on the Web. The Excel plug-in is free and open-source. Codes for developers, as well as a guide to install the plug-in for users are available at https://github.com/RealEstateCore/ExcelRDF [23].

### 5.4 Dissemination principle

#### 5.4.1 OH Consensus Report Annotation Checklist (OH-CRAC)

The comparison of surveillance results provided in sector-specific reports is currently limited because most of these results are defined in a sector-specific context. Sometimes sector-specific reports lack some background information that report readers need to know in order to correctly interpret the surveillance results. Such missing metadata can therefore reduce the value of sector-specific surveillance activities within an OH setting. To overcome this issue, we propose the application of the OH Consensus Report Annotation Checklist (OH-CRAC) to sector-specific reports. OH-CRAC allows the mapping of existing OHS meta-information from different sectors to a predefined consensus meta-information framework that is compliant to the Generic Statistical Business Process Model (GSBPM) standard [24]. OH-CRAC also supports report creators to go through their report and evaluate whether there is any meta-information missing that provides relevant OH context to their report. In this way, future surveillance reports can be improved to support cross-sector comparability and completeness. The OH-CRAC can be completed using an interactive online tool (https://aflex.vrac.iastate.edu/checklist/?t=OH-CRAC [25]) that facilitates the provisioning of meta-information in an OH-CRAC compliant format from existing text elements in a given report (draft). The completed OH-CRAC can then easily be attached to the final surveillance data report, and furthermore be used as metadata for datasets that are created during the corresponding surveillance activity.

#### 5.4.2 Template for One Health reporting of antimicrobial resistance and use in food-borne zoonosis

Many countries carry out surveillance on antimicrobial use (AMU) in human and animals and antimicrobial resistance (AMR) in animals, food and humans. This generates up to five different data streams from surveillance activities and components. In most countries, multiple agencies are involved and data is often reported either by individual sectors, e.g. human and animals, by datatype, e.g. AMU or AMR, or even by individual data streams, e.g. AMU in animals. All these data streams and their derived interpretations serve a purpose and are aimed at stakeholders benefiting from and applying control measures according to the surveillance outputs. However, reporting to the OH objective is much rarer and utilisation of all data-streams and all information for an OH purpose is rare. Addressing the OH objective and communication surveillance aimed at this specifically, is likely to promote the OH agenda and principles to stakeholders and help in combating AMR.

Two examples were generated during the ORION project. In those examples, different data streams from AMR and AMU surveillance reporting for both Campylobacter and Salmonella were integrated to achieve the OH objective of ensuring effective treatment in humans. The examples demonstrate use of all five data streams to address this surveillance objective and fully integrate the data analyses across the data streams, where it was required to support decision support with an OH perspective. The examples were published in DANMAP 2019, Chapter 6 [26]. Generic templates for analysing AMR and AMU data for zoonoses in an OH context will be included in the OHS Codex.

## 6 Conclusions and future perspectives

This paper describes six tools in the OHS Codex. However, the Codex will continue to evolve as further tools, solutions and resources to enhance the implementation of OH in surveillance are developed and can then be added to the OHS Codex (https://oh-surveillance-codex.readthedocs.io/en/latest/index.html [10]), as they become available.

In Europe, the OH paradigm is well accepted and understood by stakeholders, institutions and experts involved in OH related activities. However, achieving progress along this paradigm remains a constant challenge. Community resources that help overcome the many intrinsic resistances linked with changing established procedures and systems, are therefore of specific value for OH practitioners. For Europe, the OHS Codex represents the first non-normative, continuously updateable framework for resources supporting the adoption of the OH approach. With its scope on promoting surveillance data exchange and interpretation between European OH sectors, it complements the high-level principles and global scale guidelines of the Tripartite Zoonoses Guide [4]. Unique features of the OHS Codex are:

• It is an open, logically structured, web-based community inventory of resources that can easily be accessed and used as well as maintained and updated by the European OH community.
• It contains reports from national OH efforts from different countries that provide success stories, but also illustrate limitations or challenges. The collection of tools and examples concludes that neither improved cross-sector collaboration nor availability of advanced tools guarantees progress towards a specific OH objective.
• It acknowledges that no single strategy can be prescribed to all EU countries on how to carry out OHS in practice. The level of cross-sector connectedness in the various steps of the surveillance pathway varies across countries, and even within countries, for different zoonotic pathogens.
• It will be listed as an OH resource in the future Surveillance and Information Sharing Operational Tool (SISOT) of WHO/FAO/OIE (release date envisaged for 2021) [27].

Yet the OHS Codex is subject to certain limitations:

• It is centred on resources that help to improve the harmonization and interpretation of surveillance data. An extension into other OH areas requires the revision of the OHS Codex purpose and scope statements and the addition of new principles to the framework.
• Even though the OHS Codex is implemented as a clearly structured, web-based resource, it can be difficult to find the right information that addresses a user-specific OH problem. This is also linked to the fact that tools and resources need to be placed under one specific principle, even if they support multiple ones.

The exploitation, evolvement and continuous update of the OHS Codex by OH professionals require continuous investments into its dissemination and curation, and a commitment of organisation(s) to “feel responsible”. For the duration of the OHEJP project (2018–2022), such investments are assured as well as the development and implementation of a long-term maintenance strategy that aligns with the overarching OHEJP sustainability plan.

## Abbreviations

**Table:** 

AMR	AntiMicrobial Resistance
AMU	AntiMicrobial Use
ECDC	European Centre for Disease Prevention and Control
EFSA	European Food Safety Authority
EID	Emerging Infectious Diseases
EU	European Union
FAIR	Findable, Accessible, Interoperable, Reusable
FAO	Food and Agriculture Organisation of the United Nations
GSBPM	Generic Statistical Business Process Model
NGS	Next Generation Sequencing
OH	One Health
OH-CRAC	One Health Consensus Report Annotation Checklist
OHEJP	One Health European Joint Programme
OHS	One Health Surveillance
OIE	World Organisation for Animal Health, formerly the Office International des Epizooties
ORION	One health suRveillance Initiative on harmOnization of data collection and interpretatioN
WHO	World Health Organisation

## Author contributions

Matthias Filter: Conceptualization; Writing - Original Draft; Writing - Review & Editing; Project administration; Funding acquisition

Tasja Buschhardt: Conceptualization; Visualization; Writing - Review & Editing

Fernanda Dórea: Conceptualization; Writing - Original Draft; Writing - Review & Editing; Funding acquisition

Estibaliz Lopez de Abechuco: Writing - Original Draft; Visualization; Writing - Review & Editing

Taras Günther: Visualization; Resources

Esther M. Sundermann: Writing - Review & Editing

Jörn Gethmann: Writing - Original Draft

Johanna Dups-Bergmann: Writing - Review & Editing

Karin Lagesen: Writing - Original Draft

Johanne Ellis-Iversen: Conceptualization; Writing - Original Draft; Writing - Review & Editing

## Declaration of Competing Interest

None.

## Appendix A Supplementary data

Manuscript glossary Image 1

## References

[1] Jeggo M., Mackenzie J.S., Atlas R.M., Maloy S. (2014). Defining the future of One Health. One Health.

[2] Bordier M., Delavenne C., Nguyen D.T.T., Goutard F.L., Hendrikx P. (2019). One health surveillance: a matrix to evaluate multisectoral collaboration. Front. Veterin. Sci..

[3] Stärk K.D., Arroyo Kuribreña M., Dauphin G., Vokaty S., Ward M.P., Wieland B., Lindberg A. (2015). One Health surveillance - More than a buzz word?. Prevent. Veterin. Med..

[4] WHO, FAO, OIE (2019). Taking a Multisectoral, One Health Approach: A Tripartite Guide to Addressing Zoonotic Diseases in Countries. http://www.fao.org/3/ca2942en/ca2942en.pdf.

[5] (2015). One Health Global Network Webportal. http://www.onehealthglobal.net.

[6] (2021). One Health European Joint Programme. https://onehealthejp.eu/.

[7] (2020). The OHEJP ORION Project. https://onehealthejp.eu/jip-orion/.

[8] FAO, WHO Codex Alimentarius. http://www.fao.org/fao-who-codexalimentarius/en/.

[9] ORION (2020). Deliverable-JIP1-D1.1 Report on requirement analysis for OH Surveillance Codex (ORION).

[10] Bordier M., Uea-Anuwong T., Binot A., Hendrikx P., Goutard F.L. (2018). Characteristics of one health surveillance systems: a systematic literature review. Prevent. Veterin.ary Medicine.

[11] European Statistical System (2019). Quality Assurance Framework of the European Statistical System v2.0. https://ec.europa.eu/eurostat/documents/64157/4392716/ESS-QAF-V1-2final.pdf/bbf5970c-1adf-46c8-afc3-58ce177a0646.

[12] ORION Consortium (2020). One Health Surveillance Codex Document. https://oh-surveillance-codex.readthedocs.io/en/latest/index.html.

[13] ORION Consortium (2020). One Health Surveillance Codex - Guidelines for OHS Codex contributors. https://oh-surveillance-codex.readthedocs.io/en/latest/6a-guidelines-contributors.html.

[14] ORION Consortium (2020). One Health Surveillance Codex - Collaboration Principle. https://oh-surveillance-codex.readthedocs.io/en/latest/2-the-collaboration-principle.html.

[15] ORION Consortium (2020). One Health Surveillance Codex - Knowledge Principle. https://oh-surveillance-codex.readthedocs.io/en/latest/3-knowledge-principle.html.

[16] ORION Consortium (2020). One Health Surveillance Codex - Data Principle. https://oh-surveillance-codex.readthedocs.io/en/latest/4-the-data-principle.html.

[17] ORION Consortium (2020). One Health Surveillance Codex - Dissemination Principle. https://oh-surveillance-codex.readthedocs.io/en/latest/5-the-dissemination-principle.html.

[18] ORION Consortium (2020). OHEJP Glossary. https://foodrisklabs.bfr.bund.de/ohejp-Glossary/.

[19] European Food Safety Authority (2013). Standard sample description ver. 2.0. EFSA J..

[20] Smith B., Ashburner M., Rosse C., Bard J., Bug W., Ceusters W., Goldberg L.J., Eilbeck K., Ireland A., Mungall C.J., Leontis N., Rocca-Serra P., Ruttenberg A., Sansone S.-A., Scheuermann R.H., Shah N., Whetzel P.L., Lewis S., The O.B.I.C. (2007). The OBO foundry: coordinated evolution of ontologies to support biomedical data integration. Nat. Biotechnol..

[21] Data Driven Surveillance (2018). Health Surveillance Ontology (HSO). http://datadrivensurveillance.org/health-surveillance-ontology-hso/.

[22] Wilkinson M.D., Dumontier M., Aalbersberg I.J., Appleton G., Axton M., Baak A., Blomberg N., Boiten J.-W., da Silva Santos L.B., Bourne P.E., Bouwman J., Brookes A.J., Clark T., Crosas M., Dillo I., Dumon O., Edmunds S., Evelo C.T., Finkers R., Gonzalez-Beltran A., Gray A.J.G., Groth P., Goble C., Grethe J.S., Heringa J., t Hoen P.A.C., Hooft R., Kuhn T., Kok R., Kok J., Lusher S.J., Martone M.E., Mons A., Packer A.L., Persson B., Rocca-Serra P., Roos M., van Schaik R., Sansone S.-A., Schultes E., Sengstag T., Slater T., Strawn G., Swertz M.A., Thompson M., van der Lei J., van Mulligen E., Velterop J., Waagmeester A., Wittenburg P., Wolstencroft K., Zhao J., Mons B. (2016). The FAIR Guiding Principles for scientific data management and stewardship. Scientific Data.

[23] Hammar K. (2020). ExcelRDF Documentation. https://github.com/RealEstateCore/ExcelRDF.

[24] United Nations Economic Commission for Europe, Statistical office of the European Union (2019). Organisation for Economic Co-operation and Development Work Session on Statistical Metadata Generic Statistical Business Process Model (GSBPM) v5.1. https://statswiki.unece.org/display/GSBPM/GSBPM+v5.1.

[25] Iowa State University'’s Virtual Reality Applications Center (VRAC) (2020). RIGOR: Reporting Interface for Guidelines on Research, OH-CRAC. https://aflex.vrac.iastate.edu/checklist/?t=OH-CRAC.

[26] Korsgaard H.B.E., Ellis-Iversen J.E., Wolf Sönksen U.E., Skovgaard S.E., Hendriksen R.S., Borck Høg B., Ellis-Iversen J., Petersen C.K., Boel J., Dalby T., Hammerum A.M., Hansen F., Hasman H., Henius A.E., Hoffmann S., Ilan M.B., Kaya H., Kjerulf A., Torpdahl M., DANMAP (2019). Use of Antimicrobial Agents and Occurrence of Antimicrobial Resistance in Bacteria From Food Animals, Food and Humans in Denmark 2020. https://www.danmap.org/-/media/arkiv/projekt-sites/danmap/danmap-reports/danmap-2019/danmap_2019.pdf?la=en.

[27] FAO, OIE, WHO (2019). FAO, OIE, and WHO Launch a Guide for Countries on Taking a One Health Approach to Addressing Zoonotic Diseases. http://www.fao.org/ag/againfo/home/en/news_archive/2019_TZG.html.

