# Supplementary material for: One Health Surveillance Codex: promoting the adoption of One Health solutions within and across European countries
Source: One Health. 2021 Mar 5;12:100233. doi: 10.1016/j.onehlt.2021.100233 (PMC7994538; doi:10.1016/j.onehlt.2021.100233)
Supplement: Supplementary file 1 — Manuscript glossary [file mmc1.docx]

**Manuscript glossary**

The following table contains all the relevant OHEJP Glossary terms (<https://foodrisklabs.bfr.bund.de/ohejp-glossary/> [1]) present in the manuscript. This table was generated using the so-called Glossaryfication service [1], a web-based “text processing” tool that automatically searches for the terms that are contained in the OHEJP Glossary within any user-provided text document. Through an interactive user-interface the user can select those terms and corresponding definitions that match the intended meaning within the user-provided text document. A list of terms and definitions, along with their sector classification, abundance in the provided text document (frequency) and references is generated.

| **OHEJP Term** | **Definition** | **Sector classification** | **Reference** |
| --- | --- | --- | --- |
| [Data](http://data.d4science.org/ctlg/ORIONKnowledgeHub/748-d8b214f5-fb62-4787-9230-2e9f87b0ec7c) | Facts, measurements, recordings, records, or observations about the world collected by scientists and others, with a minimum of contextual interpretation. Data may be in any format or medium taking the form of writings, notes, numbers, symbols, text, images, films, video, sound recordings, pictorial reproductions, drawings, designs or other graphical representations, procedural manuals, forms, diagrams, work flow charts, equipment descriptions, data files, data processing algorithms, or statistical records. | Shared Definition | [2] |
| [Environment](http://data.d4science.org/ctlg/ORIONKnowledgeHub/7ee28f83-3dc4-4d68-ac6d-e3a0a0dfe5d4) | All that which is external to the individual, including physical, biological, social, cultural and other factors. | Shared Definition | [3] |
| [FAIR data](http://data.d4science.org/ctlg/ORIONKnowledgeHub/18-2312c17a-430f-4862-a849-6538fdafe9be) | Findable, accessible, interoperable and re-usable data, i.e. data that are managed according to the FAIR Guiding Principles. The FAIR Principles describe distinct considerations for contemporary data publishing environments with respect to supporting both manual and automated deposition, exploration, sharing, and reuse. | Shared Definition | [4] |
| [Food safety](http://data.d4science.org/ctlg/ORIONKnowledgeHub/473-41724a88-d9ae-4f09-a2a1-482cf99a6b94) | Assurance that food will not cause harm to the consumer when it is prepared and/or eaten according to its intended use. | Shared Definition | [5] |
| [Knowledge](http://data.d4science.org/ctlg/ORIONKnowledgeHub/752-f451b7ab-b799-4b84-8c78-a8fbed7ffe51) | A combination of data and information, to which is added expert opinion, skills, and experience, resulting in a valuable asset that aids decision making. In organizational terms, knowledge is generally thought of as being know-how, applied information, information with judgment, or the capacity for effective action. Knowledge may be tacit, explicit, individual, and/or collective. It is intrinsically linked to people | Shared Definition | [6] |
| [Metadata](http://data.d4science.org/ctlg/ORIONKnowledgeHub/669-902a27b0-d75a-4511-866f-ff70b08e238d) | Literally, "data about data"; data that defines and describes the characteristics of other data, used to improve understanding of data and data-related processes. | Shared Definition | [7] |
| [Multisectoral](http://data.d4science.org/ctlg/ORIONKnowledgeHub/761-93f53c79-b99b-4f39-a07e-2161c0206851) | Means that more than one sector are working together (e.g. on a joint program or response to an event), but does not imply that all relevant sectors are working together | Shared Definition | [8] |
| [One Health](http://data.d4science.org/ctlg/ORIONKnowledgeHub/5a01249c-750e-4f70-a2b6-ebb466078476) | A concept that recognizes the optimal health of people as being connected to the health of animals and the environment. The collaborative effort of multiple disciplines working locally, nationally, and globally to attain optimal health for people, animals, and our environment. A concept that became an approach and then a movement. | Shared Definition | [3] |
| [One Health](http://data.d4science.org/ctlg/ORIONKnowledgeHub/626-95a72367-fde0-44c2-bdf2-e6322e32827e) | One Health is an approach to designing and implementing programmes, policies, legislation and research in which multiple sectors communicate and work together to achieve better public health outcomes. The areas of work in which a One Health approach is particularly relevant include food safety, the control of zoonoses and combatting antibiotic resistance. | Shared Definition | [9] |
| [One Health concept](http://data.d4science.org/ctlg/ORIONKnowledgeHub/bf778774-8e3c-4281-9879-df52991162ee) | A worldwide strategy for expanding interdisciplinary collaborations and communications in all aspects of health care for humans, animals and the environment. | Shared Definition | [3] |
| [ORION](http://data.d4science.org/ctlg/ORIONKnowledgeHub/814-e50c13c8-c0b3-4fcb-8b70-1ec5deca9831) | One health surveillance initiative on harmonization of data collection and interpretation. A Joint Integrative Project within the One Health EJP. | Shared Definition | [10] |
| [Pathogen](http://data.d4science.org/ctlg/ORIONKnowledgeHub/575-33bc7992-6f20-4e96-bb5b-799e7022ad8c) | Organism (e.g. bacterium, virus and parasite) that can cause disease. | Shared Definition | [11] |
| [Public health](http://data.d4science.org/ctlg/ORIONKnowledgeHub/e45aa7b7-c1cc-4d90-806d-99e7070c1ba6) | The art and science of dealing with the protection and improvement of community health by organized community effort and including preventive medicine and sanitary and social health. The science of protecting and promoting the Health of individuals and the communities where they live. | Shared Definition | [3] |
| [Risk](http://data.d4science.org/ctlg/ORIONKnowledgeHub/993-0008ec29-e24d-4b24-bf90-41a9d260dba0) | A function of the likelihood that a zoonotic disease event or contaminant may occur and the magnitude of the impact if it were to occur. | Shared Definition | [8] |
| [Sector](http://data.d4science.org/ctlg/ORIONKnowledgeHub/845-aba040a2-6a4e-4e6e-b219-c4ce6e332814) | A distinct part or branch of a nation’s sociological, economic, or political society or a sphere of activity such as human health, animal health, or environment. | Shared Definition | [8] |
| [Stakeholders](http://data.d4science.org/ctlg/ORIONKnowledgeHub/cb6e8751-c333-4b6c-9b22-1603188a0afc) | People or groups who have an involvement or interest in some system, including beneficiaries, providers and funders. | Shared Definition | [3] |
| [Surveillance](http://data.d4science.org/ctlg/ORIONKnowledgeHub/c4302944-03aa-4d38-b305-675527e108bb) | Consists of procedures developed in response to a risk and carried out to support subsequent actions. Data collection and record keeping to track the emergence and spread of disease: causing organisms (incl. antibiotic: resistant bacteria). | Shared Definition | [3] |
| [Surveillance pathway](http://data.d4science.org/ctlg/ORIONKnowledgeHub/861-eb18758f-e124-45f5-be77-022c5c5c45a5) | Describes the steps in the surveillance i.e. design adjustment and optimization of the surveillance programme/system; sample collection; laboratory analysis; data transfer and collation; data analysis and interpretation; outcome communication; response prioritization | Shared Definition | [12] |
| [Zoonosis](http://data.d4science.org/ctlg/ORIONKnowledgeHub/316-7ac2ed77-6c19-4a50-93be-d10e7e4a19cd) | Any disease and/or infection that is naturally transmissible directly or indirectly between animals and humans | Shared Definition | [13] |

**References**

[1] ORION Consortium, OHEJP Glossary, <https://foodrisklabs.bfr.bund.de/ohejp-Glossary/>, 2020 (accessed 4 November 2020).

[2] Information Consortia Advancing Standards in Research Administration, Data validation, <https://dictionary.casrai.org/Data_validation>, 2019 (accessed 17 May 2019).

[3] European Network for Neglected Vectors and Vector-Borne Infections, EurNEgVEc One Health Dictionary, <http://www.eurnegvec.org/publications/other/EurNegVecDictionary.pdf> (accessed 23 September 2019).

[4] M. D. Wilkinson, et al., The FAIR Guiding Principles for scientific data management and stewardship, (2016). 10.1038/sdata.2016.18.

[5] Commission Codex Alimentarius. General Principles of Food Hygiene CAC/RCP 1-1969, 2004, <http://www.fao.org/fao-who-codexalimentarius/sh-proxy/en/?lnk=1&url=https%253A%252F%252Fworkspace.fao.org%252Fsites%252Fcodex%252FStandards%252FCXC%2B1-1969%252FCXP_001e.pdf>.

[6] O. Serrat, Glossary of Knowledge Management, Springer Singapore, 2017. <https://doi.org/10.1007/978-981-10-0983-9_120>.

[7] Consortia Advancing Standards in Research Administration Information, Metadata, <https://dictionary.casrai.org/Data_validation>, 2019 (accessed 17 May 2019).

[8] FAO, OIE, WHO. Taking a Multisectoral, One Health Approach: A Tripartite Guide to Addressing Zoonotic Diseases in Countries 2019, <http://www.fao.org/3/ca2942en/ca2942en.pdf>.

[9] Organization World Health, WHO One Health, <https://www.who.int/features/qa/one-health/en/>, 2019 (accessed 29 April 2019).

[10] ORION Consortium, ORION definition, <https://aginfra.d4science.org/catalogue-aginfra?path=/dataset/814-e50c13c8-c0b3-4fcb-8b70-1ec5deca9831>, 2020 (accessed 5 November 2020).

[11] Authority European Food Safety, EFSA Glossary of Terms, <https://www.efsa.europa.eu/en/glossary-taxonomy-terms>, 2019 (accessed 6 June 2019).

[12] ORION Consortium, Surveillance pathway definition, <https://aginfra.d4science.org/catalogue-aginfra?path=/dataset/861-eb18758f-e124-45f5-be77-022c5c5c45a5>, 2020 (accessed 5 November 2020).

[13] Council of the European Union European Parliament. Directive 2003/99/EC of the European Parliament and of the Council of 17 November 2003 on the monitoring of zoonoses and zoonotic agents, amending Council Decision 90/424/EEC and repealing Council Directive 92/117/EEC, 2003, <http://data.europa.eu/eli/dir/2003/99/oj>.
